# A C1qDC Protein (HcC1qDC6) with Three Tandem C1q Domains Is Involved in Immune Response of Triangle-Shell Pearl Mussel (*Hyriopsis cumingii*)

**DOI:** 10.3389/fphys.2017.00521

**Published:** 2017-07-21

**Authors:** Ying Huang, Lei Wu, Min Jin, Kaimin Hui, Qian Ren

**Affiliations:** ^1^Jiangsu Key Laboratory for Biodiversity and Biotechnology and Jiangsu Key Laboratory for Aquatic Crustacean Diseases, College of Life Sciences, Nanjing Normal University Nanjing, China; ^2^State Key Laboratory Breeding Base of Marine Genetic Resource, Third Institute of Oceanography, State Oceanic Administration (SOA) Xiamen, China; ^3^Co-Innovation Center for Marine Bio-Industry Technology of Jiangsu Province Lianyungang, China

**Keywords:** *Hyriopsis cumingii*, C1qDC protein, RNAi, antibacterial activity, innate immunity

## Abstract

C1q-domain-containing (C1qDC) proteins are a family of proteins with a globular C1q (gC1q) domain and participate in several immune responses. In this study, a *C1qDC* gene was identified from the triangle-shell pearl mussel *Hyriopsis cumingii* (designated as *HcC1qDC6*). This gene has a full-length cDNA of 1782 bp and an open reading frame of 1,335 bp that encodes a 444-amino acid polypeptide containing three gC1q domains. *HcC1qDC6* contains at least five exons and four introns. The mRNA transcripts of *HcC1qDC6* were found to have the highest expression levels in the mantle tissue. The expression levels in the mantle and hepatopancreas were significantly upregulated by *Staphylococcus aureus* and *Vibrio parahaemolyticus* challenges. Moreover, knockdown of *HcC1qDC6* inhibits the expression of two immune-related genes (*tumor necrosis factor* and *whey acidic protein*). The recombinant proteins of C1q1, C1q2, and C1q3 all exhibit a binding activity against seven bacterial species and directly bind to peptidoglycan and lipopolysaccharide. The results indicate that HcC1qDC6 is involved in the innate immunity of *H. cumingii*.

## Introduction

C1q is a subcomponent of the classical pathway of the complement system and a major connecting link between the innate and antibody-mediated acquired immunity (Gaboriaud et al., 2003; Kishore et al., 2004; Ghebrehiwet et al., 2012). The C1q-domain-containing (C1qDC) proteins are a large family of proteins characterized by a globular C1q (gC1q) domain of around 140 amino acids in the C-terminus with 8 highly conserved residues (positions F124, F142, N142, F160, G166, Y168, F242, and G244 in human C1q B chain; Kishore and Reid, 2000; Roumenina et al., 2011). The gC1q domain has a 10-stranded β-sandwich jelly roll topology containing two 5-stranded antiparallel β-sheets (Gaboriaud et al., 2003). The β-strands are strongly conserved relative to their orientation and size, while the loops connecting the β-strands exhibit a significant variability (Kishore and Reid, 2000). C1qDC proteins are categorized as follows. C1q/C1q-like proteins contain the amino acid repeat of Gly–Pro–X, forming the collagen region in the N-terminus, whereas the globular head C1q (ghC1q) proteins only have a short N-terminal amino acid sequence with signal peptide, coiled coil, and other sequence motifs (Carland and Gerwick, 2010).

The following are substantial *C1qDC* genes identified from vertebrates: 52 C1qDC gene models in zebrafish (Mei and Gui, 2008), 29 C1qDC gene models in mice (Yuzaki, 2008), and 32 C1qDC gene models in humans (Tang et al., 2005). Many C1qDC proteins have also been identified from platyhelminthes to cephalochordates. For instance, 50 *C1qDC* genes were reported in the amphioxus *Branchiostoma floridae* (Huang et al., 2008; Yu et al., 2008), 7 in the purple sea urchin (Hibino et al., 2006), 168 in Mediterranean mussel *Mytilus galloprovincialis* (Gerdol et al., 2011), and 321 in oyster *Crassostrea gigas* (Zhang et al., 2012). Invertebrate C1qDC proteins with C1q domain are unable to interact with other downstream proteases, resulting in the inactivation of complement pathway (Carland and Gerwick, 2010; Liu et al., 2014). Evidence suggests that invertebrate C1qDC proteins are involved in various immune responses, including microbial recognition (Kong et al., 2010; Wang et al., 2012a; Jiang et al., 2015), agglutination (Kong et al., 2010; Wang et al., 2012a), phagocytosis promotion (Wang et al., 2012b), and cell migration (Tahtouh et al., 2009). For example, two novel gC1q-domain-containing proteins, namely, AiC1qDC-1 and AiC1qDC-2, from bay scallop *Argopecten irradians* agglutinate various microorganisms and bind different pathogen-associated molecular patterns (PAMPs; Kong et al., 2010; Wang et al., 2012a). CgC1qDC-1 from *C. gigas* has high binding specificity and affinity to lipopolysaccharides (LPS). This protein serves as a pattern recognition receptor (PRR) and opsonin involved in the immune response against invading Gram-negative bacteria (Jiang et al., 2015). CfC1qDC, from Zhikong scallop *Chlamys farreri*, enhances the phagocytic activity of hemocytes toward the Gram-negative bacteria *Escherichia coli* and also bind to human heat-aggregated IgG (Wang et al., 2012b). Recently, the results of the genome and transcriptome survey revealed that some C1qDC proteins with multiple gC1q domains are found in mollusk (oyster *C. gigas* and *M. galloprovincialis;* Zhang et al., 2012; Liu et al., 2014). However, the role and mechanism of C1q domains in invertebrates innate immunity are still not yet well understood.

The triangle-shell pearl mussel *Hyriopsis cumingii* uses innate immunity to fight against a variety of pathogens (Zasloff, 2002; Rolff and Siva-Jothy, 2003). The expansion of *C1qDC* and other immune-related genes allows the mussels to adapt to different environments. Previous studies reveal that five *C1qDC* genes are involved in the innate immunity of *H. cumingii* (Huang et al., 2016; Zhao et al., 2016). In the present study, a *C1qDC* gene *HcC1qDC6* was detected from *H. cumingii*. The *HcC1qDC6* transcript is upregulated under bacterial stimulation and its protein binds to different microorganisms and carbohydrates, preventing bacterial infections.

## Materials and methods

### Experimental animals and microbes

A total of 250 healthy *H. cumingii* with an average weight of 15 g, ~1 year old, were purchased from an aquaculture farm in Wuhu City, Anhui Province, China. The freshwater mussels were maintained in aerated freshwater and allowed to acclimate at room temperature for 7 days before processing.

LPS (*E. coli* serotype 055:B5) and PGN (*Staphylococcus staphylolyticus*) were purchased from Sigma (St. Louis, MO, USA). *Staphylococcus aureus, Micrococcus luteus, Bacillus subtilis, Vibrio anguillarum, E. coli* (maintained and available in our laboratory), and *Vibrio parahaemolyticus* (ATCC 17802, Microbial Culture Collection Center, Beijing, China) bacteria were grown in Luria–Bertani (LB) at 37°C. *Aeromonas hydrophila* (ATCC 7966, Microbial Culture Collection Center, Beijing, China) was grown in LB broth and incubated at a temperature of 28°C.

### Immune challenge, total RNA isolation, and first-strand cDNA synthesis

A total of 100 μl of *S. aureus* (3 × 10^7^ cells) or *V. parahaemolyticus* (3 × 10^7^ cells) was injected into the adductor muscles of *H. cumingii* using a 1-mL sterile syringe. Phosphate-buffered saline (PBS; 0.14 M NaCl, 3 mM KCl, 8 mM Na_2_HPO_4_, and 1.5 mM KH_2_PO_4_; pH 7.4) was used as a control. At 2, 6, 12, and 24 h post-bacterial or PBS injection, the hepatopancreas or mantles from three *H. cumingii* were mixed for RNA extraction. Hemocytes, hepatopancreas, gills, and mantles from three healthy mussels were extracted and mixed for RNA extraction and further tissue distribution analysis. All experiments were repeated three times.

Pure RNA High-Purity Total RNA Rapid Extraction Kit (Spin-Column; Bioteke, Beijing, China) was used to isolate the total RNA from above samples based on the manufacturer's protocol. First-strand cDNA synthesis for quantitative real-time (qRT) polymerase chain reaction (PCR) analysis was conducted using the PrimeScript® First-Strand cDNA Synthesis Kit (Takara, Dalian, China) with the Oligo-d(T) Primer. Approximately 5 μg of the total RNA obtained from the hepatopancreas were reverse transcribed via the Clontech SMARTer™ RACE cDNA Amplification Kit (Takara, Dalian, China) with 5′-CDS Primer A and SMARTerIIA oligos (5′-RACE Ready cDNA) and 3′-CDS Primer A (3′-RACE-Ready cDNA). The detailed procedures were performed based on the manufacturer's instructions.

### Cloning of the full-length cDNA encoding *HcC1qDC6*

The data analysis of the hepatopancreas transcriptome of *H. cumingii* (unpublished data) revealed a sequence of *C1qDC* gene (*HcC1qDC6*). Then, the 5′ and 3′ fragments of *HcC1qDC6* were amplified using gene-specific primers (*HcC1qDC6*-F: 5′-GCGTGCGAATGCCAAATAGCGGAG-3′ and *HcC1qDC6*-R: 5′-CCACTCCGCTATTTGGCATTCGCAC-3′) and a Universal Primer A Mix (UPM). The PCR was performed in accordance with the following procedures: 1 cycle at 94°C for 2 min; 30 cycles at 94°C for 30 s, 68°C for 30 s, and 72°C for 3 min; and 1 cycle at 72°C for 2 min using the Clontech Advantage 2 PCR Kit (Takara, Dalian, China). The full-length *HcC1qDC6* cDNA was obtained by overlapping the 5′ and 3′ fragments.

### Sequence analysis of *HcC1qDC6*

The cDNA sequence of *HcC1qDC6* was analyzed using BLAST (http://blast.ncbi.nlm.nih.gov/Blast.cgi), and the deduced protein sequences were obtained with ExPASy (http://web.expasy.org/). Signal peptide and domain organization were predicted with SMART (http://smart.embl-heidelberg.de/). The theoretical isoelectric point (*p*I) and molecular weight (MW) were determined using ExPASy (http://web.expasy.org/compute_pi/). MEGA 5.05 and GENDOC were used to align HcC1qDC5 and HcC1qDC6. MEGA 5.05 was utilized to phylogenetically analyze the relationship of HcC1qDC6 and other C1qDC proteins from *H. cumingii* and other species (Kumar et al., 2008).

### Extraction of genomic DNA and amplification of introns of *HcC1qDC6*

First, genomic DNA was extracted from the gills of healthy mussels by using NucleoSpin Tissue (Clontech). Next genome walking was performed using the Universal GenomeWalker 2.0 Kit (Clontech) to amplify the genome sequences of *HcC1qDC6*. The first round of PCR was done using the gene-specific forward primer *HcC1qDC6*-walk-F1: 5′-GTGCCCTGCCGAAGGAGTTTACAAGTT-3′ and primer AP1 (5′-GTAATACGACTCACTATAGGGC-3′). The digestive genomic DNA was used as template in the first round PCR. The second round of PCR was then conducted using primers *HcC1qDC6*-walk-F2: 5′-CACAGAAGGAGATGCGATTTTCGTCAA-3′ and AP2: 5′-ACTATAGGGCACGCGTGGT-3′. In order to walk toward 5′ end, nested PCRs were performed using sequence-specific reverse primers (*HcC1qDC6*-walk-R1: 5′-CCAGAGAGAACCCAAAGCAACATCCAA-3′, *HcC1qDC6*-walk-R2: 5′-CGTGTCTTGACGAAAATCGCATCTCCT-3′) and AP1 and AP2 primers. Finally, a pair of gene-specific primers (g*HcC1qDC6*-F: 5′-TTCCAGGATGATTGCTTTCTCTGTCGG-3′, g*HcC1qDC6*-R: 5′-TTAAGGTGTGGACGAGACAAGGTAACC-3′) were designed to amplify partial genome sequences using undigested genomic DNA as template. Above 3 fragments were overlapped to obtain the genome sequences of *HcC1qDC6*. The above procedures were performed according to the manufacturer's protocols.

### Small interfering RNA (siRNA)-mediated RNA interference assay

Based on the sequence of *HcC1qDC6*, the siRNA was specifically designed by BLOCK-iT™ RNAi Designer to target *HcC1qDC6* and then synthesized *in vitro* with a commercial kit based on the manufacturer's instructions (Takara, Japan). The siRNA used was *HcC1qDC6*-siRNA (5′-GGUAUGGAUCCACAGGUAA-3′), and the siRNA sequence was scrambled to generate the control *HcC1qDC6*-siRNA-scrambled (5′-UGUGAAGAUGGAACGUACC-3′).

RNA interference (RNAi) assay was performed by injecting 40 μg of siRNA into the adductor muscles of each *H. cumingii*. Approximately 20 μg of siRNA or siRNA-scrambled cells and *V. parahaemolyticus* (3 × 10^7^ cells) were injected into a mussel at a volume of 100 μL per mussel. At 16 h after the injection, 20 μg of siRNA or siRNA-scrambled cells (100 μL/mussel) were injected into the same mussel. At the same time, 100 μL of *V. parahaemolyticus* was injected into the mussel as a positive control. For each group, 27 mussels were used. At 24, 36, and 48 h after the last injection, the gills and mantles from three mussels were collected. The experiment was repeated three times on each sample.

### qRT-PCR assay

The tissue distribution and expression patterns of *HcC1qDC6* gene at an mRNA level were analyzed through qRT-PCR using the primers *HcC1qDC6*-RT-F: 5′-GCGTGCGAATGCCAAATAG-3′ and *HcC1qDC6*-RT-R: 5′-ATGGACAGGGGCCGTAAAA-3′. The gills and mantle tissues of *HcC1qDC6* knockdown mussels were tested to identify the transcriptional levels of *tumor necrosis factor HcTNF* and *whey acidic protein HcWAP*. The primers *HcTNF*-RT-F: 5′-ATTCCTTCCTGACATACAAAACCGA-3′ and *HcTNF*-RT-R: 5′-AGAAAGTGAAGAGTGGAGGCGAGAT-3′; *HcWAP*-RT-F: 5′-TGTAATGTTGACGGGAGTG-3′, and *HcWAP*-RT-R: 5′-CTGTTTTGTTTTGATGGCT-3′ were used in the test. β*-Actin* was amplified as an internal control using *Hc-actin*-RT-F: 5′-GTGGCTACTCCTTCACAACC-3′ and *Hc-actin*-RT-R: 5′-GAAGCTAGGCTGGAACAAGG-3′. The methods utilized were based from the previous study (Huang et al., 2013). Each sample used for qRT-PCR analysis was prepared in triplicates. The 2^−ΔΔCT^ method was used to calculate the expression levels of genes (Livak and Schmittgen, 2001). All data were given as mean ± standard error (S.E). Unpaired sample *t*-test was applied, in which *P* < 0.05 was considered statistically significant.

### Expression and purification of three C1q domains of HcC1qDC6 protein

Three pairs of primers with *EcoR* I sites in the forward primers and *Xho* I sites in the reverse primers (rC1q1-ex-F: 5′-TATCGGATCCGAATTCGCAAATACACAAGATGATGTC-3′ and rC1q1-ex-R: 5′-GGTGGTGGTGCTCGAGCATTCGCACGCCGCTGAACG-3′; rC1q2-ex-F: 5′-TATCGGATCCGAATTCTCAAATGGATATAAGGACGAC-3′ and rC1q2-ex-R: 5′-GGTGGTGGTGCTCGAGCTCTCTGATTATTAGGGCAC-3′; rC1q3-ex-F: 5′-TATCGGATCCGAATTCAACATACCGCTGAGTTCCAGG-3′ and rC1q3-ex-R: 5′-GGTGGTGGTGCTCGAGTGTGGACGAGACAAGGTAAC-3′) were used to amplify the cDNA fragments of three C1q domains of HcC1qDC6. Both fragments and pET-30a(+) vector were digested with *EcoR* I and *Xho* I, and the purified DNA fragments were ligated into the linearized pET-30a(+) vector. The plasmids were transformed into *E. coli* BL21 (DE3) competent cells for recombinant expression. The recombinant C1q proteins with an N-terminal His tag were purified using His-Bind resin chromatography (Novagen, USA). The procedure was done in accordance with the manufacturer's instructions (Huang et al., 2014). Moreover, sodium dodecyl sulfate polyacrylamide gel electrophoresis (SDS-PAGE, 12.5%) is the method used to detect the purified protein.

### Binding of recombinant proteins to microorganisms

The binding activity of recombinant proteins to bacteria was analyzed through a microbial binding assay with slight modifications (Huang et al., 2014). Three Gram-positive bacteria, namely, *S. aureus, M. luteus*, and *B. subtilis*, and four Gram-negative bacteria, namely, *A. hydrophila, V. parahaemolyticus, V. anguillarum*, and *E. coli*, were utilized in the bacterial binding assay. These bacteria were cultured overnight in LB broth, washed three times with tris-buffered saline (TBS), centrifuged at 6000 rpm for 5 min at room temperature, and then resuspended in TBS. The purified recombinant proteins (600 μg/mL, 500 μL) were added, incubated with microorganisms (2 × 10^8^ cells/mL), and gently rotated for 1 h at room temperature. Microorganisms were washed four times with TBS, subsequently resuspended in 50 μL of TBS, and analyzed using 12.5% of SDS-PAGE.

### Polysaccharide-binding assay

An enzyme-linked immunosorbent assay (ELISA) was performed to detect the direct binding of rC1q proteins to LPS and peptidoglycan (PGN; Zhang et al., 2011). In summary, sugar was initially dissolved in water at a concentration of 80 μg/mL. Each well of a microtiter plate was then coated with 25 μL of sugar solution. Afterwards, the microtiter plate was incubated at 37°C overnight and heated at 60°C for 30 min. Each well was blocked with 200 μL of bovine serum albumin (BSA, 600 μg/mL) at 37°C for 2 h and then washed four times with tris-buffered saline with Tween 20 (TBST). The purified proteins with gradient dilution (0–30 μg/mL in TBS contains 0.1 mg/mL BSA) were added to the wells and incubated at 28°C for 3 h. The wells were washed four times; a total of 100 μL of anti-His antiserum (1/2,000 diluted in 0.1 mg/mL BSA) was then added and incubated at 37°C for 1 h. Each well was washed four times and then incubated with 100 μL of peroxidase-conjugated goat anti-rabbit IgG (1/10,000 diluted) for 1 h at 37°C. The plate was washed four times with TBST and developed with 0.01% 3,3′,5,5′-tetramethylbenzidine (Sigma). Approximately 2 M H_2_SO_4_ was utilized to prevent the reaction. The absorbance was read and noted at 450 nm. His-tag protein ADP ribosylation factor (rArf) was used in this assay as control. The assays were performed in triplicates.

## Result

### Identification of the cDNA encoding *HcC1qDC6*

**Figure 1:**
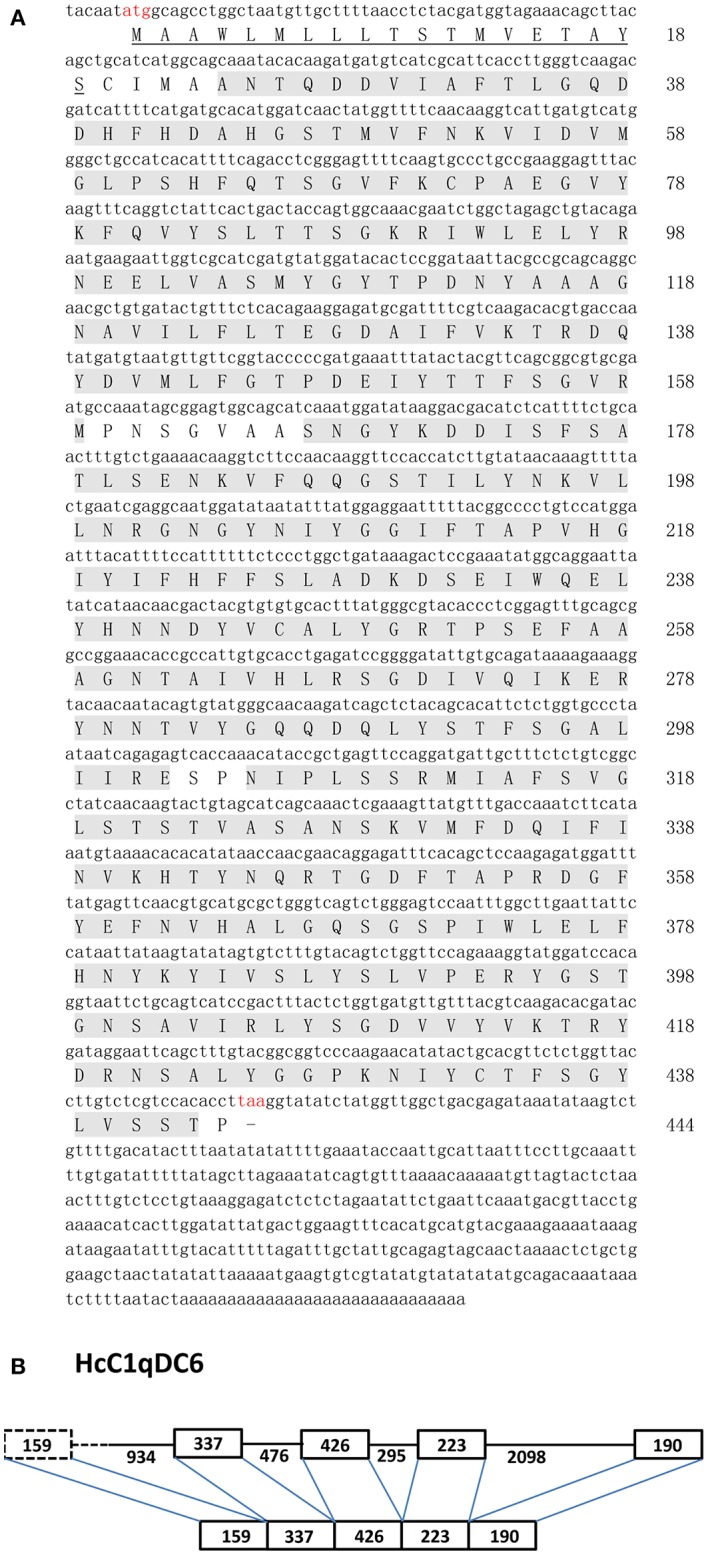
**(A)** Nucleotide and deduced amino acid sequences of *HcC1qDC6* from *H. cumingii* (above). Signal peptide sequences are underlined, and three C1q domains are shadowed. **(B)** Genomic structure of *HcC1qDC6* (below).

The full sequence of *HcC1qDC6* (GenBank accession no. MF038002) was 1782 bp in length. This sequence contains 1335 bp of open reading frame encoding a 444-amino-acid protein, 6 bp of 5′-untranslated terminal region (UTR), and a 441-bp of 3′ UTR with a poly(A) tail (Figure 1A). *HcC1qDC6* was analyzed using the SMART program. Results show that the gene contains a signal peptide of 19-amino-acid residues and three C1q domains (amino acids 24–159, 167–302, and 305–443). The MW and *p*I of HcC1qDC6 were 49.5 kDa and 5.76, respectively. The MWs of rC1q1 to rC1q3 were 15.2, 15.4, and 15.5 kDa, respectively, and rC1q1 to rC1q3 have *p*I values of 4.70, 5.71, and 9.21, respectively.

### Genomic organization of *HcC1qDC6*

The partial genome sequence of *HcC1qDC6* contained at least five exons interrupted by four introns. Because 5′ end of the *HcC1qDC6* genomic sequence was not fully amplified, the length of the four exons (337, 426, 223, and 190 bp) and three introns (476, 295, and 2,098 bp) were only determined. The exon–intron boundaries of *HcC1qDC6* genomic sequence are GT and AG at 5′ and 3′ splice sites (Figure 1B and Figure S1).

### HcC1qDC6 homologous analysis

**Figure 2:**
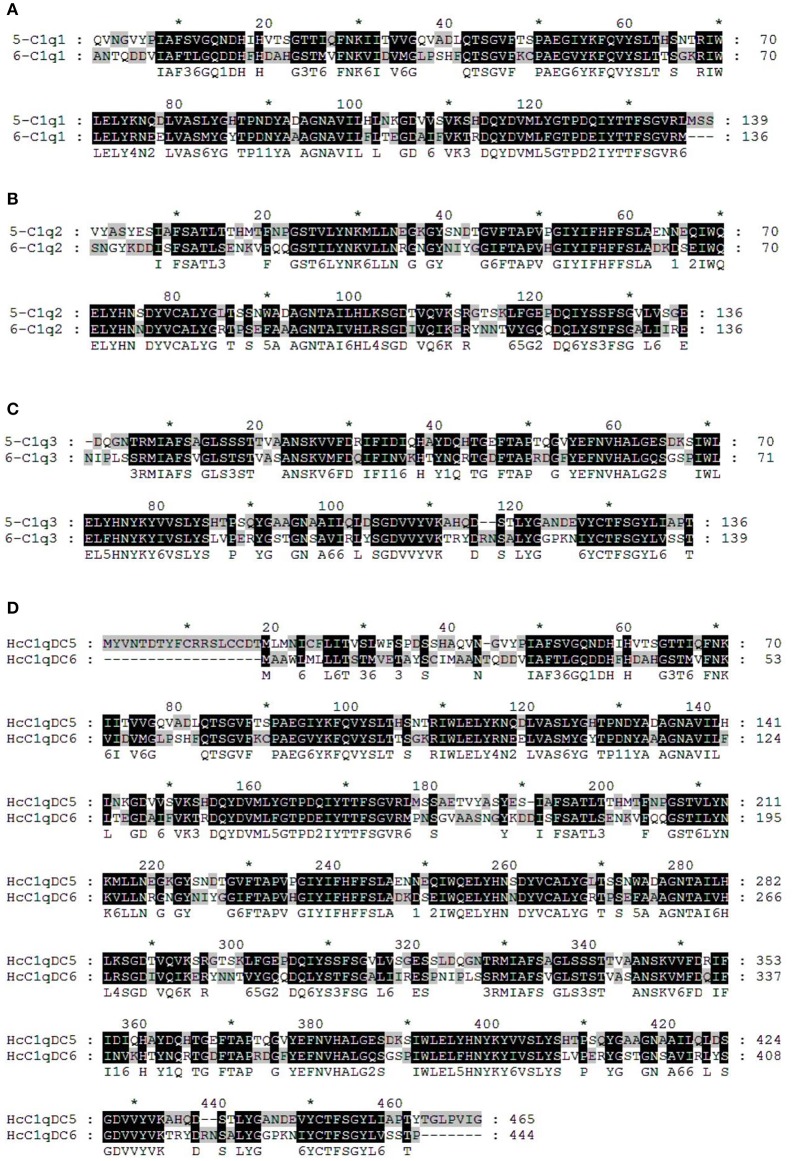
Multiple alignment analyses of the first C1q domain **(A)**, the second C1q domain **(B)**, the third C1q domain **(C)**, and the full length amino acid sequences **(D)** of HcC1qDC5 and HcC1qDC6.

**Figure 3:**
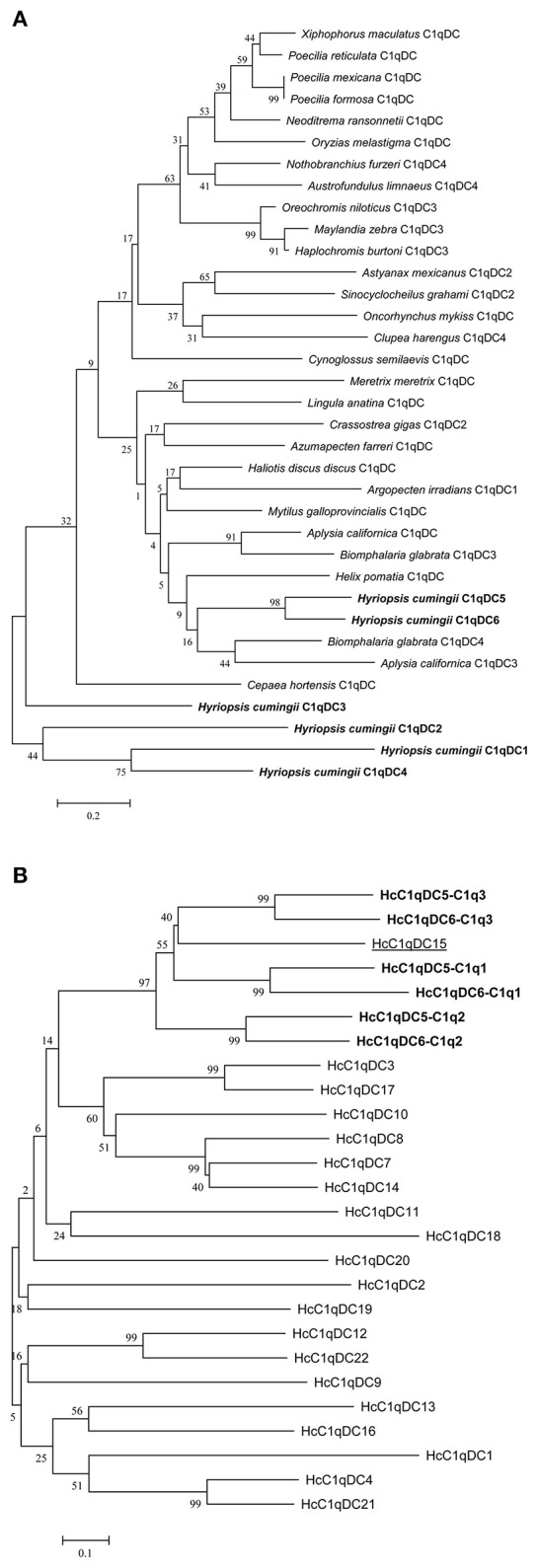
**(A)** Phylogenetic tree analysis of HcC1qDC6 and other representative C1qDC proteins. *C. gigas* C1qDC2, Accession No. EKC25478.1; *B. glabrata* C1qDC4, Accession No. XP_013091954.1; *Haliotis discus discus* C1qDC, Accession No. ABO26662.1; *M. galloprovincialis* C1qDC, Accession No. CBX41690.1; *A. californica* C1qDC, Accession No. XP_005112559.2; *Cepaea hortensis* C1qDC, Accession No. CAD83837.1; *B. glabrata* C1qDC3, Accession No. XP_013081758.1; *Azumapecten farreri* C1qDC, Accession No. AGI44588.1; *A. irradians* C1qDC1, Accession No. ADD17343.1; *Neoditrema ransonnetii* C1qDC, Accession No. BAI40067.1; *Oryzias melastigma* C1qDC, Accession No. AEA51019.1; *Astyanax mexicanus* C1qDC2, Accession No. XP_007230354.1; *Helix pomatia* C1qDC, Accession No. ABF00124.1; *Maylandia zebra* C1qDC3, Accession No. XP_014264880.1; *Cynoglossus semilaevis* C1qDC, Accession No. NP_001290212.1; *Xiphophorus maculatus* C1qDC, Accession No. XP_005816097.1; *Meretrix meretrix* C1qDC, Accession No. ADX99230.1; *Lingula anatina* C1qDC, Accession No. XP_013398186.1; *Poecilia mexicana* C1qDC, Accession No. XP_014841021.1; *A. californica* C1qDC3, Accession No. XP_012940093.1; *Oreochromis niloticus* C1qDC3, Accession No. XP_003454798.2; *Poecilia formosa* C1qDC, Accession No. XP_007574024.1; *Nothobranchius furzeri* C1qDC4, Accession No. XP_015801801.1; *Poecilia reticulata* C1qDC, Accession No. XP_008420785.1; *Oncorhynchus mykiss* C1qDC, Accession No. CDQ68953.1; *Austrofundulus limnaeus* C1qDC4, Accession No. XP_013878127.1; *Haplochromis burtoni* C1qDC3, Accession No. XP_005952871.1; *Clupea harengus* C1qDC4, Accession No. XP_012680903.1; *Sinocyclocheilus grahami* C1qDC2, Accession No. XP_016096745.1. **(B)** Phylogenetic tree analysis of the C1q domains of HcC1qDC6 and other *H. cumingii* C1qDC proteins.

BLASTP analysis showed that HcC1qDC6 shares 49% identity with complement C1q-like protein 2 from *C. gigas* and 45% identity with putative complement C1q-like protein 4 from *Biomphalaria glabrata*. The C1q1, C1q2, and C1q3 domains of HcC1qDC6 showed higher identity with three corresponding C1q domains of HcC1qDC5 (Figures 2A–C). The full length amino acid sequences of HcC1qDC6 and HcC1qDC5 were highly conserved (Figure 2D). The constructed phylogenetic tree presents the HcC1qDC6 protein initially clustered together with HcC1qDC5 from *H. cumingii* and then joined with C1qDC4 from *B. glabrata* and C1qDC3 from *Aplysia californica* (Figure 3A). Another phylogenetic tree using C1q domains of different C1qDCs (20 single C1q domain-containing C1qDCs and 2 multiple C1q domain-containing C1qDCs) from *H. cumingii* was also constructed. Results showed that C1q1, C1q2, and C1q3 of HcC1qDC5 were clustered with C1q1, C1q2, and C1q3 of HcC1qDC6, respectively. Only a single C1q domain-containing C1qDC (HcC1qDC15) were grouped with C1q domains of HcC1qDC5 and HcC1qDC6 (Figure 3B).

### Tissue distribution and mRNA expression analysis of *HcC1qDC6*

**Figure 4:**
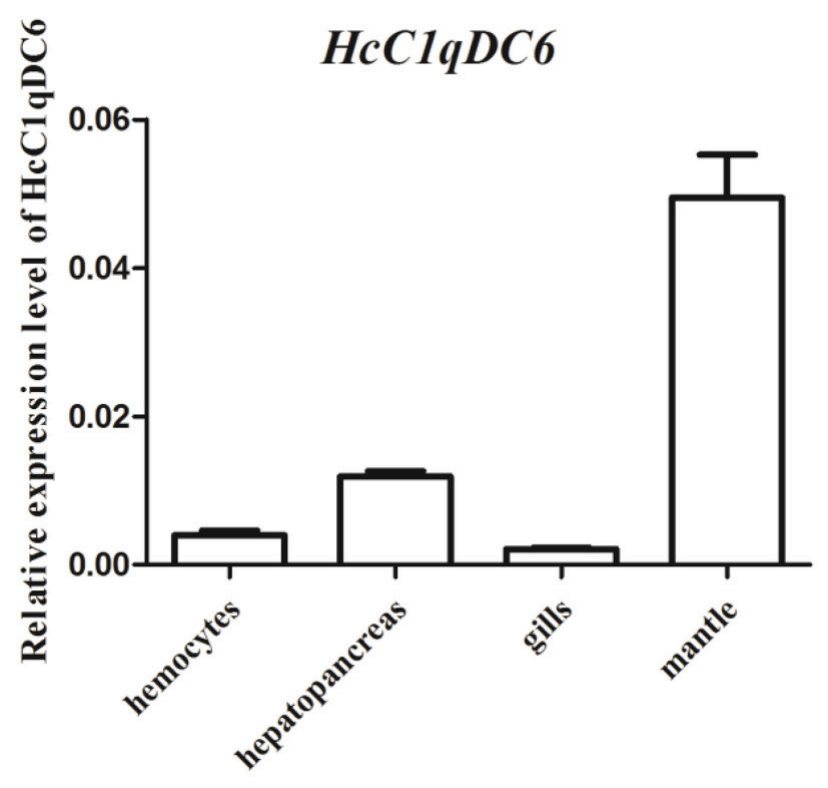
qRT-PCR analysis of *HcC1qDC6* in the hemocytes, hepatopancreas, gills, and mantles of *H. cumingii*.

Tissue distribution analysis was conducted through qRT-PCR. Results revealed that *HcC1qDC6* was expressed in all tested tissues, such as, mantles, hepatopancreas, hemocytes, and gills (Figure 4).

**Figure 5:**
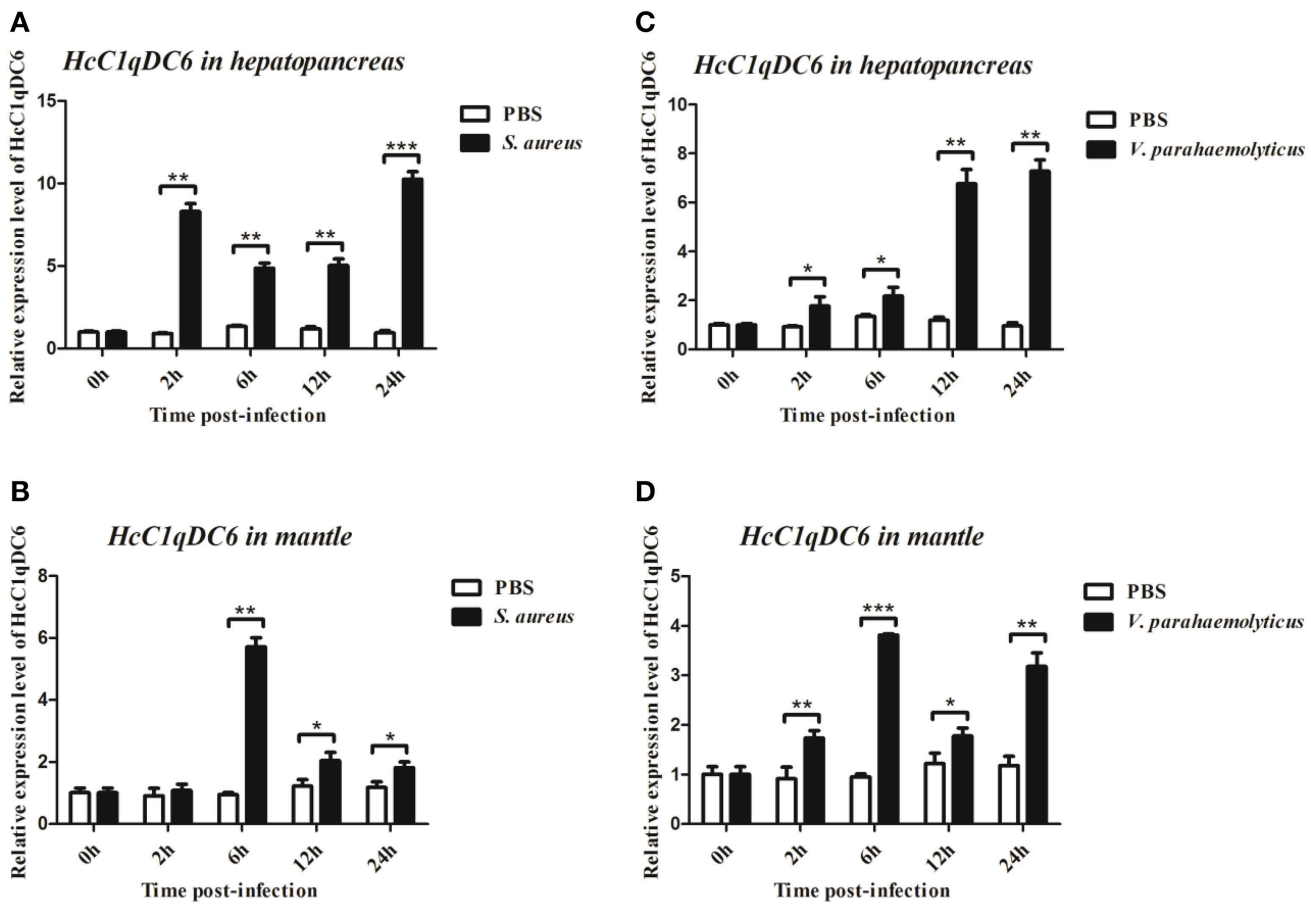
Time course analysis of *HcC1qDC6* expression pattern in the hepatopancreas and mantles of *H. cumingii* under *S. aureus* or *V. parahaemolyticus* challenge at 2, 6, 12, and 24 h. Asterisks indicate significant differences (^*^P < 0.05, ^**^P < 0.01, ^***^P < 0.001) when compared with that of the control.

qRT-PCR was also used to monitor the mRNA expression of *HcC1qDC6* transcripts in the hepatopancreas and mantles of mussels stimulated by two bacteria (Figure 5). In *S. aureus*-stimulated group, the *HcC1qDC6* transcription level in the hepatopancreas was initially upregulated at 2 h, slightly downregulated from 6 to 12 h, and finally increased to the highest level at 24 h. *HcC1qDC6* in the mantles was significantly upregulated at 6 h after stimulation and then gradually decreased to the original level. In the *V. parahaemolyticus* stimulated group, *HcC1qDC6* mRNA in the hepatopancreas was slowly increased at 2 and 6 h and then quickly upregulated from 12 to 24 h. After a significant increase at 6 h post-stimulation, the mRNA expression of *HcC1qDC6* in the mantles decreased at 12 h and then gradually increased at 24 h. In the control group, no significant change in *HcC1qDC6* expression was observed during the entire experiment after PBS injection.

### Expression analysis of *HcTNF* and *HcWAP* after knockdown of *HcC1qDC6*

**Figure 6:**
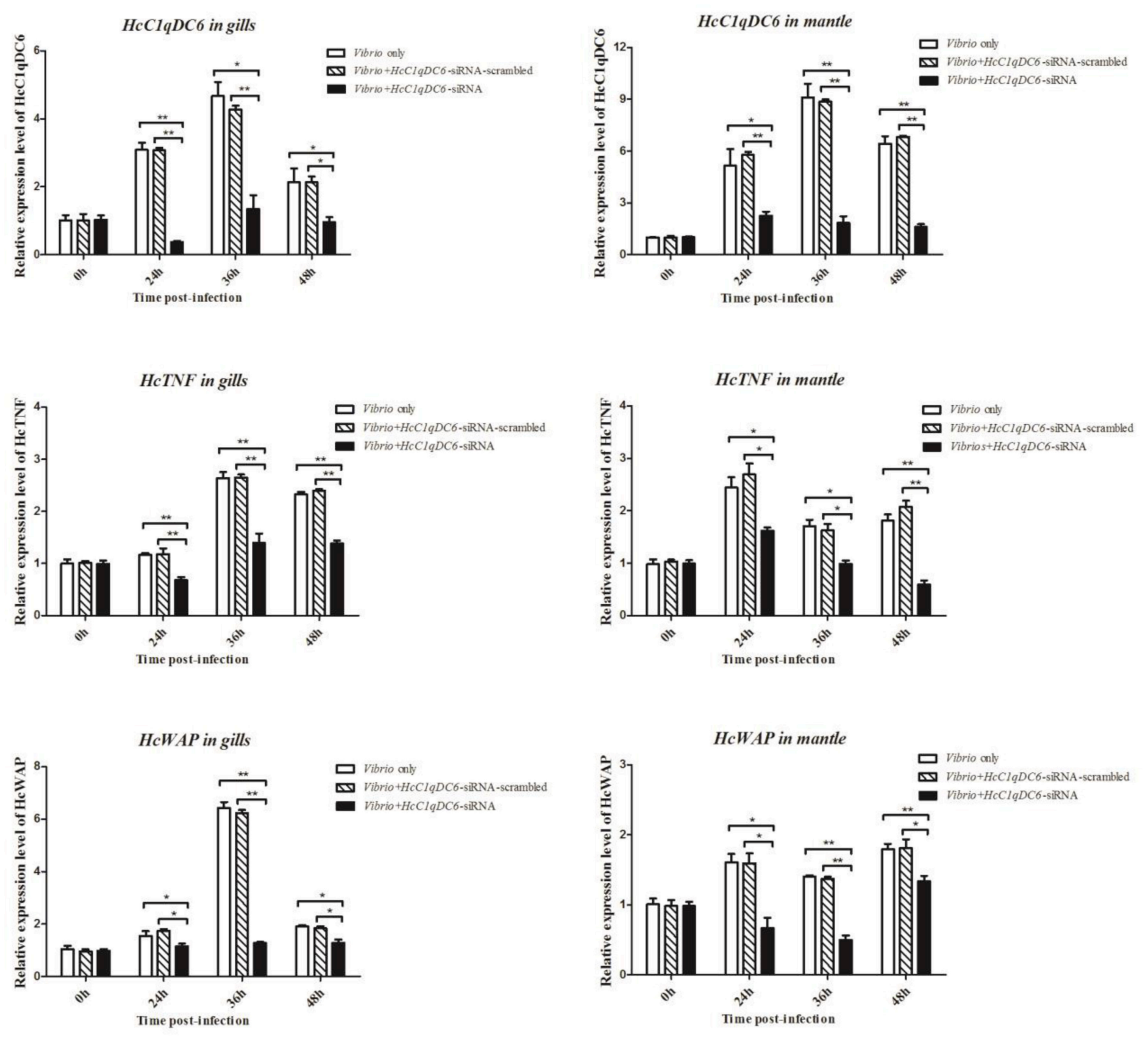
*HcTNF* and *HcWAP* expression analyses of the *HcC1qDC6* knocking down *H. cumingii* challenged by *V. parahaemolyticus*. Asterisks indicate significant differences (^*^P < 0.05, ^**^P < 0.01) when compared with the control. Error bars represent ± *SD* of the three independent PCR amplifications and quantifications.

The siRNA interference group showed a reduced *HcC1qDC6* expression levels after 24, 36, and 48 h of *V. parahaemolyticus* challenge as compared with that of the control groups (*V. parahaemolyticus* only and siRNA-scrambled group; Figure 6). C1qDC protein functions as a PRR in innate immunity. The expression levels of effector genes, such as, *TNF* and *WAP*, were analyzed after knockdown of *C1qDC6*. The expression levels of *HcTNF* and *HcWAP* decreased in the mussels with *HcC1qDC6* knockdown as compared with that of the control groups (*V. parahaemolyticus* only and siRNA-scrambled group; Figure 6).

### Recombinant expression of rC1q1 to rC1q3 in *E. coli*

**Figure 7:**
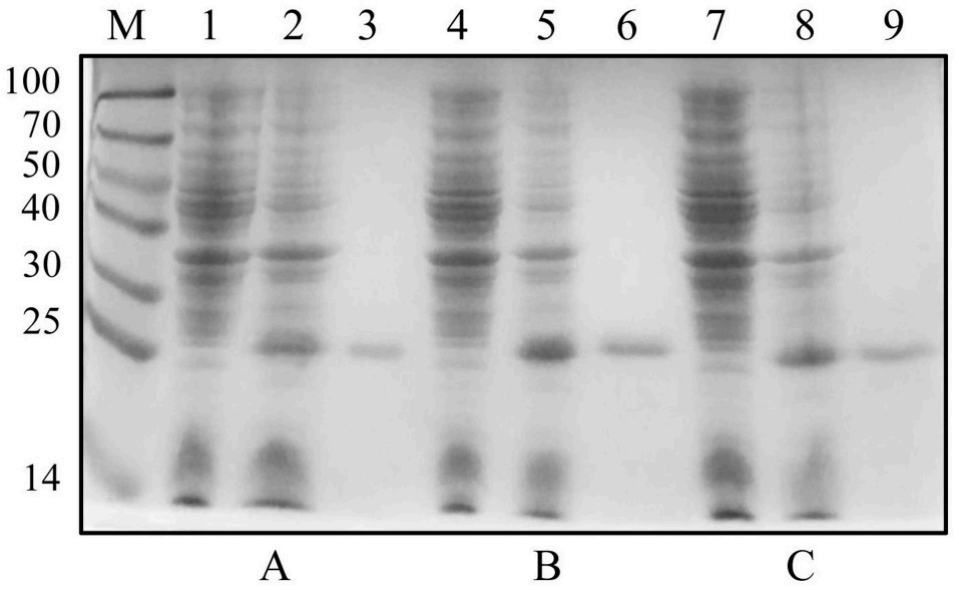
Expression and purification levels of recombinant three C1q domains. **(A–C)** show expression and purification levels of C1q1, C1q2, and C1q3. Lane M, protein marker; lanes 1, 4, and 7, crude protein extracts of *E. coli* without induction; lanes 2, 5, and 8, crude protein extracts of *E. coli* with IPTG induction; lanes 3, 6, and 9, purified recombinant proteins by His-Bind resin chromatography.

The plasmids pET-30a-rC1q1, pET-30a-rC1q2, and pET-30a-rC1q3 were transformed into *E. coli* BL21 (DE3). Cell lysates were then analyzed using SDS-PAGE after IPTG induction. Results showed that rC1q1 to rC1q3 were successfully expressed and purified (Figure 7).

### Microbial binding activity of rC1qs

**Figure 8:**
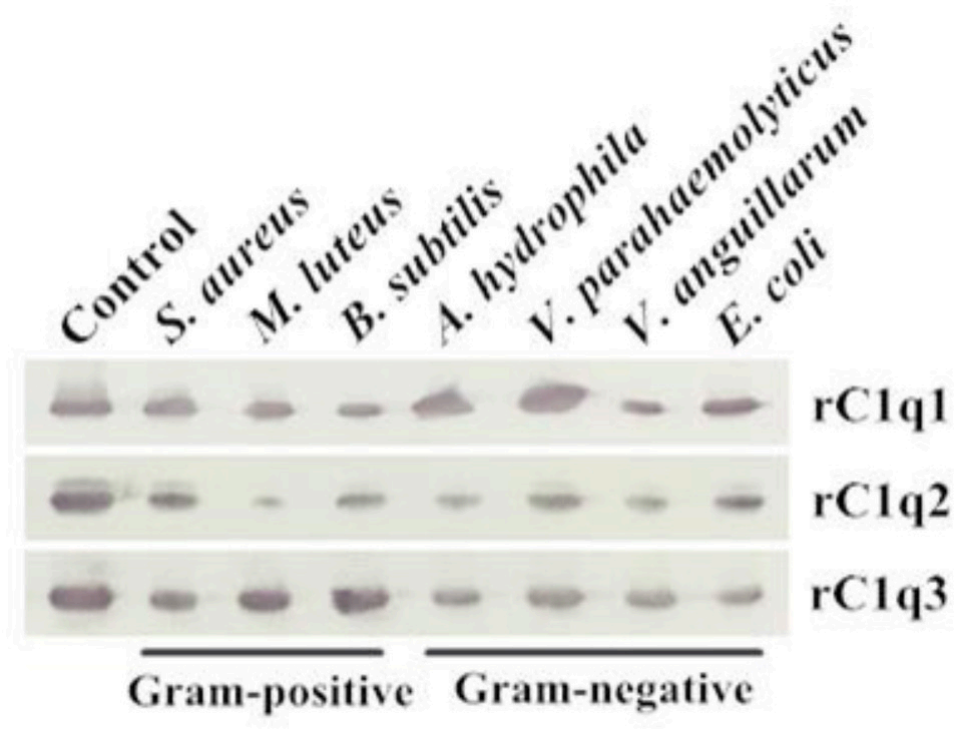
Direct binding of rC1q1 to rC1q3 to seven bacterial strains. The recombinant proteins were incubated with microorganisms first and then washed four times with PBS. Binding activity was confirmed using Western blot with anti-His rabbit mAb against recombinant C1q proteins with His-tag.

A bacterial binding assay was performed to determine the binding capacity of three recombinant C1q domains (Figure 8). Results suggested that rC1q1 to rC1q3 can bind to all tested microorganisms, including Gram-positive bacteria (*S. aureus, M. luteus*, and *B. subtilis*) and Gram-negative bacteria (*A. hydrophila, V. parahaemolyticus, V. anguillarum*, and *E. coli*). rC1q1 exhibits a strong binding activity against *A. hydrophila* and *V. parahaemolyticus*, whereas rC1q2 strongly binds to *S. aureus* and *V. parahaemolyticus* but has a weak binding activity toward *M. luteus*. rC1q3 shows stronger binding activity toward Gram-positive bacteria than Gram-negative bacteria.

### PAMPs binding activity of rC1qs

**Figure 9:**
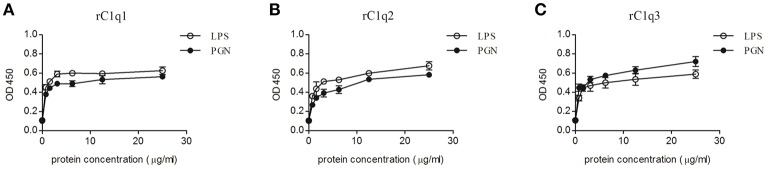
ELISA assay that detects the binding of rC1q1, rC1q2, or rC1q3 to LPS and PGN using anti-His antiserum. The data shown are the mean ± SE obtained from three individual experiments.

Optical density (OD) value is used to measure the binding activity of rC1q1, rC1q2, and rC1q3 to different PAMPs. Figure 9 shows an OD value of 450 nm. The recombinant rC1q proteins exhibited different affinity to LPS and PGN and a dose-dependent binding ability. The rArf group showed an OD value of 0.1, thereby indicating that rArf could not bind any tested PAMPs (data not shown).

## Discussion

Pathogen recognition is the first and crucial step in innate immunity, discriminates the self from the potentially harmful non-self, and activates a series of immune responses (Yang et al., 2011). C1qDC proteins can recognize a remarkable variety of non-self ligands, including certain bacteria, viruses, parasites, and mycoplasmas, through the gC1q domain (Kishore et al., 2004; Ghebrehiwet et al., 2012). The current studies revealed that some invertebrate C1qDC proteins function as PRRs and bind pathogens directly by engaging PAMPs (Kishore et al., 2004; Bohlson et al., 2007; Ghebrehiwet et al., 2012). In this study, a novel multidomain C1qDC protein (HcC1qDC6) was found from *H. cumingii*. Unlike most identified invertebrate C1qDC proteins, three gC1q domains in HcC1qDC6 were identified. In the phylogenetic tree, HcC1qDC6 and HcC1qDC5 were clustered together. These two C1qDCs both belong to multiple C1q domain-containing proteins. The HcC1qDC6 is categorized as a ghC1q protein when the N-terminal has no collagen region. However, HcC1qDC6 contains a signal peptide in the N-terminal, which controls the entry of proteins to the secretory pathway both in eukaryotes and prokaryotes. This peptide is involved in a wider range of immune responses (Nielsen et al., 1997).

The *HcC1qDC6* mRNA is constitutively expressed in the mantles, hepatopancreas, hemocytes, and gills, which is similar to that found in *HcC1qDC1* to *HcC1qDC5* from *H. cumingii* (Huang et al., 2016; Zhao et al., 2016), *AiC1qDC-1* and *AiC1qDC-2* from *A. irradians* (Kong et al., 2010; Wang et al., 2012a), *CfC1qDC* from *C. farreri* (Jiang et al., 2015), and *L-C1qDC-1* from *Lethenteron camtschaticum* (Pei et al., 2016). The *HcC1qDC6* transcript is highly expressed in the mantle and mantle tissue plays a crucial role in pathogen prevention and clearance (Wang et al., 2013). *HcC1qDC6* is also expressed in the hepatopancreas. Hepatopancreas is equivalent to the fat body of insects and plays an important function in the humoral immune responses of invertebrate (Gross et al., 2001). After the mussels were challenged by the Gram-positive bacteria *S. aureus* or Gram-negative bacteria *V. parahaemolyticus*, expression levels of *HcC1qDC6* in both the hepatopancreas and mantles increased within 24 h. This finding indicates that *HcC1qDC6* is stimulated by pathogens and involved in the immune response against infections.

In invertebrates, C1qDC proteins function as PRRs and directly bind to pathogens by engaging a broad range of PAMPs, triggering a series of immune processes (Medzhitov and Janeway, 2002; Bohlson et al., 2007). For example, AmphiC1q1 protein directly interacts with LPS (Yu et al., 2008). CfC1qDC strongly binds to LPS, PGN, β-glucan, and poly I:C (Wang et al., 2012b). AiC1qDC-2 is able to recognize various PAMPs and exhibits strong agglutination to the Gram-negative bacteria *E. coli* and *V. anguillarum*, the Gram-positive bacteria *B. subtilis*, and the fungi *Pichia pastoris* (Wang et al., 2012a). In this study, all three gC1q domains of HcC1qDC6 directly bind to a broad range of PAMPs, including PGN exposed from Gram-positive bacteria and LPS from Gram-negative bacteria. Three gC1q domains strongly bind to Gram-negative and Gram-positive bacteria. In addition, HcC1qDC proteins also exhibited a good binding ability (Huang et al., 2016; Zhao et al., 2016).

In this study, the expression levels of two immune-related genes *HcTNF* and *HcWAP* were analyzed in *HcC1qDC6*-silenced mussels challenged with *V. parahaemolyticus*. TNF superfamily genes have been found in invertebrates such as, shrimp and mollusk (Wang et al., 2012; Gao et al., 2015). CgTNF-1 enhanced the hemocyte apoptosis and phagocytosis and elevated the antibacterial activity of oyster *C. gigas* (Sun et al., 2014). WAP is involved in the host immune defense with antibacterial and proteinase inhibitory activities (Li et al., 2013). The *HcTNF* and *HcWAP* transcripts were downregulated with *HcC1qDC6* knockdown. Furthermore, *HcC1qDC6* directly or indirectly regulates the *HcTNF* and *HcWAP* expression levels.

Overall, multiple C1q domain-containing C1qDC proteins (*HcC1qDC6*) were identified from *H. cumingii*. The expression levels of *HcC1qDC6* changes in response to bacterial infections. Furthermore, the direct binding ability of *HcC1qDC6* to different bacteria and PAMPs indicates pattern recognition of the innate immunity of *H. cumingii*.

## Ethics statement

We declare that appropriate ethical approval and licenses were obtained during our research.

## Author contributions

YH, LW, MJ, and KH carried out the experiments. YH and QR designed the experiments and analyzed the data. YH and QR wrote the manuscript. All authors gave final approval for publication.

### Conflict of interest statement

The authors declare that the research was conducted in the absence of any commercial or financial relationships that could be construed as a potential conflict of interest.
