# Supplementary material for: A C1qDC Protein (HcC1qDC6) with Three Tandem C1q Domains Is Involved in Immune Response of Triangle-Shell Pearl Mussel (Hyriopsis cumingii)
Source: Front Physiol. 2017 Jul 21;8:521. doi: 10.3389/fphys.2017.00521 (PMC5519582; doi:10.3389/fphys.2017.00521)
Supplement: Supplementary file 1 [file DataSheet1.docx]

CGACGGCCCGGGCTGGTAAAAAATGGGTTACATAATAAGTTCTAATGGCTTTTCGCATGTAATACTAATCTACACATTTCAGTTTATTACCGCTTTGAAGAACAGCAAAAACTTTTCTTCTTCAGGATGTGCATAACAGTTTATGCTTAAATACAAAATATGTATTACTGGTAAAAAAAAAATCTTTAATGACGCATACGCTCAAGTTTAACACCATTTTTACAATTAACACTTATTCCACTGCAATCGTTCTTGTTACAGACAATATTCAATGATACTGTTACATATTATGTTTATGCAGCCCAAAGTCACGTACTTAAGACCGTCAAGCTACTATTAACGAATCCTCCATCCCTTTGAACGAGGTTAGATTTAGTCCGTGAACTTCCACATTATGCACGGACTTGTATTTAGATTTCGAGTTTTAGTTCTTGATAAAGCCATCACGTAACTTTACGTCAGGCTAAAAATTAAATATTATCATTGTCAAAAATGTTTTAACAAAGGCAGTAGTCGAATATCGATTTTTTTCAACGGAAACTAAACTAACATGGAAGTCTTGCTGCAAGACATGCCTTTGCGTGGTATTATATCAAAACTAACATCAGTGCTCTATATCATGTATTTAGTACCTGTTGACAAAATTACGCCAATTATGACATATAGAACGTTAAAGTCAAAGTGAGTTGTTTTTAAATATTATATTTTGCCAAGCTATTAATTTAATTACCAAACCAGATTACATTCTGTTATCGTGATAAACAAACTCTGGCCAACATGCAAATGCTAAGTCCAGACAAAATCTGGTGCTCAATAATATTTAAGTGTCAAATATTAAAAAAAAGAAACTAGGTGTGGGATGTACATTTAAAACGATATGCGTCACATATGTCTCCTTCTTTAACGATTATTGATCACAGTCTTTCCTTTATAGGTCATTGATGTCATGGGGCTGCCATCACATTTTCAGACCTCGGGAGTTTTCAAGTGCCCTGCCGAAGGAGTTTACAAGTTTCAGGTCTATTCACTGACTACCAGTGGCAAACGAATCTGACTAGAGCTGTACAGAAATGAAGAATTGGTCGCATCGATGTATGGATACACTCCGGATAATTACGCCGCAGCAGGCAACGCTGTGATACTGTTTCTCACAGAAGGAGATGCGATTTTCGTCAAGACACGTGACCAATATGATGTAATGTTGTTCGGTACCCCCGATGAAATTTATACTACGTTCAGCGGCGTGCGAATGCCAAATAGCGGAGTGGCAGGTAGCAATTCCTCTAAAATTGAAATGATATGGCAAAGTGCTAAAGTTCTCATTACGTGTTACATTTATGTAATAATTGTTTGAATATGTCTGCCTCGAGGGATTCGAAATAGCTTATTGTTTGTGTATTTCCTCAGATATTGAGATATATTAATAGTTTATTGGATGTTGCTTTGGGTTCTCTCTGGGCTTTTGTATTTCGAGTATATATTTTTTGTTTTGTTAAATAGTTTCATCTCTTGACAGAGAGCTTGGAACATTGGAAAACTTTTTCTACTACTTAATTACTTCTTTTCTTTCTTTATCTATATATGATTACCGATTTTTTAAAAACATCTCTGTCACACAGTAATCTTTTTTTTAAATCTTATATGACAACTTATATTTTTATTTTATTACCAATTTGTTTATATTAAGAAACATCAGCCTTCATCAATAGAGTTAGAAAGTCTGACATTATAATTTGTTTTCTTTCAGCATCAAATGGATATAAGGACGACATCTCATTTTCTGCAACTTTGTCTGAAAACAAGGTCTTCCAACAAGGTTCCACCATCTTGTATAACAAAGTTTTACTGAACCGAGGCAATGGATATAATATTTATGGAGGAATTTTTACGGCCCCTGTCCATGGAATTTACATTTTCCATTTTTTCTCCCTGGCTGATAAAGACTCCGAAATATGGCAGGAATTATATCATAACAACGACTACGTGTGTGCACTTTATGGGCGTACACCCTCGGAGTTTGCAGCGGCCGGAAACACCGCCATTGTGCACCTGAGATCCGGGGATATTGTGCAGATAAAAGCCAGGCAGAACAATACAGTGTATGGGCAACAAGATCAGCTCTACAGCACATTCTCTGGTGCCCTAATAATCAGAGAGTCACCAAACATACCGCGTAAGTTGATTTGTTTAATAATAAAAGTGAAAAGAAAATATATAATATAGAAGACAAATCTTAAGTTTTTCAATATCCACAATTCTGTATTGGATCTTGTCTGGAATACTGAGTGACCTTCATATGGTAGTAAAGTCATCACTTTCTGCATATAATGAAGCTTGATGTTCTAACAATATTTTAATGGATTACAAATTAAAATGTGGTATTTCAAACGATTCCTTTAACGTTACAACTGATGTGGAGGGAAACTTAAATAGCACTGCATATTCTTTCCTACGTCTCCTTTTTGCAGTGAGTTCCAGGATGATTGCTTTCTCTGTCGGCCTATCAACAAGTACTGTAGTATCAGCAAACTCGAAGGTTATGTTTGACCAAATCTTCATAAAGGTAAAACACACATATAACCAACAAACAGGAGATTTCACAGCTCCAAGAGATGGATTTTATGAGTTCAACGTGCATGCGCTGGGTCAATCTGGGAGTCCAATTTGGCTTGAATTATTCCATAATTATAAGTAAGTTTCAATATTAGCTTTTTCTTAAAACAAGTAAGAAAATAACAGCAATGACGAATATTACATTGTTATAAGTTACTTGATTTTGACTTCATCATCATCGTTACCATTGCCATCATCGTCATTATCGTGGACAATGTTTATGTTGTCATCACACCACAATTGCTATCAGCATCAGCTGTAGCTGAAATATAAATTGATTTATCTGTATGTGACTACATATTTGTATTAAGAAACGCTACAGAGTAGATTTTACATAGTTAAATTAAGCAATGTTTTTGTATTAATATTTTCACCAGTTTTCTACCACTATGAAATAAAAGTTATACAGTTTCAGTTCCAGTTTTGTGGGATTATGCAATTTACCTTATATATACAGCGGCAGGGGGCAAAAGCACCAGCCGCTGTGAAATAGAAATAACCAAAGGCCTTGTAGGAAAATACTTTAACGCGGACTATGCTGAACCATACTAGTGTTTGAGTCTGTGTTTCCTAAACAAACATCGGTAGTTTGTATTTAATCGTGTACCATGTATATATCTTTGTGGAGATACTTTTGTATGCTTTTTAATGTCAAAACGACAATAAAACAGTAGGGGAGCATACCGTTTATATTGAGAAGTAAATTACCCCTAGACATTTTAGGCAAAATATTTTAGAGCTGGGTCAGCCCATCCAAACCATTTATTGACCATGTGTTTCTGTCCCATACTAAAAAATAGAAGTGACCAAACACAAGAATTTATCAATCGAGTTGATAGATCATTTTAGCATTTAAACCTGTTGATAGATAGTTACCAAACCGTAAACCAATATTTTTTTTAAACTGACGCTCCATTTTCTAATTGATAAATTAAATGCAGTAAATGAAAAATCATGTAATTTATGGAAGCCATCCATATTAAAAAGACGGATTAATAAAACTACTTGTGTCAAATCTATGGTTTTTCGTGCACTTAGATACCTGCTGGCGGTTTTGTTCAGAACATTTCTTTTTGCGAGCGATCAAATATTTTGTAAGATACTTCACACGTATTATCATAATGCATCTTAAATGTTTATCTGATATTACTCCTACTGCAATGCGTATGTTCATTAATAAAAGAATAGTGCGAACATCTGGGTCTATTATATTTAATTGATGAGCCATGACGTTTAATATTTAGGAACCTTAACTCATTTCCTAATGAACTATGTTGCCAATGTGAATTATGATGTCTGAGGCAAGTTGACCCACTTACAGTTGTGTTTTTCATACTTACTATAATGCTTACATTTCATTCTTGCCTCCTTTTATTATTTATGTGTATGTACGTATGCTAAGTTATACCATTCAGCCTTATGTCGTAGCTGTTTTCCTTATGTAATATGGATACACGCAACGTTGGCAATAGTTACCCATCCATTAATTACATTTTTCTTTTTTCGCTTCTGCTTCTATCATACTACATTTTATGCAATTATTACACTTTCTCGTGTGCTATCAAAAATATGCAAAATAATGTTTCCAAATATACCATTAAAAACTAACCATGCAACATAACATTATATGCAGCATACTAAAGCCATTCCTTTTCATACATCTGATTCATAATGAGATCAGTCACAATTTTCCTACATGCACTTACACGTTGTGTCCTCTATTGGCACTTACACGTTGCGTTCTCTATTGGCACTTACACGTTGCGTCCTCTATTGGCACTTAAACGTTGCGTTCTCGATTGGCACTTACACGTTGCGTTCTCTATTGGCACTTACACGTTGCGTTCTCGATTGACACTTACACGTTGCGTTCTCTTTTGGCACTTACACGTTGCGTTCTCGATTGGCACTTACACGTTGCGTCCTCTATTGGCACTTTTTAAAGGACAAATCGGATTCTTGCATCAGTTAAAACTTAGTTTGGAGAGGTAATCCGTCTTGAAACTAGGGGTTACACAGAACAATAACGAACCACTTAACAATTTAAAGTTAAAACAAATATTTATTTGAACGTTCTGTAACGTTTAGCTCTTAGAAAATTGTAATAAAGTTGCTACTTCGCATTCTGTACCATAAATACGACTATTTCATTTATTTCTTTTTCCTCCTTTATCAGGTATATAGTGTCTTTGTACAGTCTGGTTCCAGAAAGGTATGGATCCACAGGTAATTCTGCAGTCATCCGACTTTACTCTGGTGATGTTGTTTACGTGAAGACACGATACGATAGGAATTCAGCTTTGTACGGCGGTCCTAAGAACATATACTGCACGTTCTCTGGTTACCTTGTCTCGTCCACACCTTAA

**Fig. S1.** Partial genomic sequence of *HcC1qDC6*.
